# How do adolescents experience a newly developed Online Single Session Sleep Intervention? A Think-Aloud Study

**DOI:** 10.1177/13591045231205475

**Published:** 2023-11-18

**Authors:** Ananya Maity, Angela W Wang, Melissa J Dreier, Vuokko Wallace, Faith Orchard, Jessica L Schleider, Maria E Loades, Jessica L Hamilton

**Affiliations:** 1 Department of Psychology, 1555 University of Bath, Bath, UK; 2 Department of Psychology, 242612 Rutgers University, New Brunswick, USA; 3 School of Psychology, 1948 University of Sussex, Brighton, UK; 4 Department of Psychology, 12301 Stony Brook University, Stony Brook, USA

**Keywords:** Qualitative, think-aloud, sleep, user experience, user experience design, single session intervention

## Abstract

**Background:**

Sleep problems are common in adolescents and have detrimental impacts on physical and mental health and daily functioning. Evidence-based treatment like cognitive behaviour therapy for insomnia (CBT-I) is often hard to access, and adolescents may not engage in and adhere to longer, clinician-delivered interventions. Brief, self-guided, and accessible sleep interventions are needed.

**Objective:**

To explore the user experience of a prototype online self-help single session sleep intervention developed for adolescents.

**Methods:**

Eleven participants aged 17–19 years (8 females, 3 males) took part in online retrospective think-aloud interviews. Participants first completed the prototype intervention independently and were then shown the intervention page by page and asked to verbalise their thoughts and experiences. Transcripts were analyzed thematically.

**Results:**

Participants found the intervention helpful. Four themes were generated - ‘Educative: Learning, but more fun’, ‘Effortless: Quicker and Easier’, ‘Personalization: Power of Choice’, and ‘Positivity: Just Good Vibes’. The theme ‘Educative: Learning, but more fun’ encompassed two sub-themes ‘Opportunity to Learn’ and ‘Aesthetics and Learning’. These themes reflected participants’ views that the intervention was educative, personalised, solution-oriented and easy to use, but could incorporate more graphics and visuals to aid in learning and could be made more effortless and positive through modifications to its design.

**Conclusions:**

Findings convey the importance of ensuring educative well-designed content, personalization, a positive tone, and ease of use while designing interventions targeting adolescents’s sleep and mental health. They also indicate areas for further developing the intervention.

Sleep problems are highly prevalent in adolescents, with most adolescents getting an inadequate amount of sleep (Marino et al., 2021). According to Short and colleagues (2013a), 60% of adolescents experience sleep problems, and the prevalence of sleep problems is up to 80% in adolescents with chronic medical, neurodevelopmental, and psychiatric conditions (Owens, 2007). A meta-analysis found that 53% of adolescents sleep for less than 8 hours per night, and 36% report difficulty falling asleep (Gradisar et al., 2011). Social, psychological, and biological factors contribute to these problems in adolescents, such as delayed timing of circadian rhythms (Hagenauer & Lee, 2013), slower build-up of sleep homeostatic pressure (Taylor et al., 2005), social, emotional, and academic stress (Short et al., 2013b), and electronic or social media use (Hale & Guan, 2015; Hamilton et al., 2020).

The consequences of poor sleep in adolescence may be far-reaching. Inadequate sleep has been associated with poor health (Reidy et al., 2016), reduced neurocognitive functioning and learning capacity (Curcio et al., 2006; Lo et al., 2016), increased daytime sleepiness and prevalence of internalizing disorders such as depression (Marino et al., 2021), and suicidal thoughts and behaviours in adolescents (Blake & Allen, 2020; Hamilton et al., 2023). This may be because sleep, arousal, and affect are all products of overlapping regulatory systems with each system impacting the other. Thus, sleep problems during key periods of maturation such as adolescence may prompt affective dysregulation (Dahl, 1996). While there is a bidirectional relationship between sleep and psychopathology, recent research indicates the potential causal role of sleep problems leading to depression (Goldstone et al., 2020; Lovato & Gradisar, 2014; Orchard et al., 2020a) and suicidal thoughts and behaviors (Liu et al., 2020). Poor sleep quality also has been linked to suicidal thoughts and behaviours in adolescents (Kearns et al., 2020), which may be due to its effects on how adolescents regulate affective responses to interpersonal events (Hamilton et al., 2023). As sleep problems are amenable to change (Blake et al., 2016), they may be an important targetable mechanism in the prevention and treatment of adolescent depression (Gee et al., 2019).

Given the importance of sleep for the mental health of adolescents, providing widely available, scalable interventions for sleep disturbances could be of great benefit in alleviating symptoms of anxiety and depression (Luik et al., 2017) and preventing suicide (Blake et al., 2018). This may be particularly important as adolescents transition towards adulthood and gain increasing autonomy and independence, including sleep-related behaviours such as bedtimes and wake-up times (Carskadon, 2011).

Several interventions addressing sleep problems, ranging from pharmacological interventions to mindfulness strategies and school-based sleep education programs, are currently available (Chung et al., 2017; Greeson et al., 2014; Owens et al., 2005). However multiple systematic meta-analytic reviews have identified Cognitive-Behavioural Therapy for Insomnia (CBT-I) as the gold standard for improving sleep (Ballesio et al., 2018; van Straten et al., 2018), and it is often the first-line treatment for adolescent sleep problems (Blake & Allen, 2020). In addition to reducing sleep problems, other meta-analyses have found that CBT-I also led to significant improvements in secondary outcomes such as anxiety and depression in both clinical and non-clinical adolescent populations (Åslund et al., 2020; Blake et al., 2017; Zetterqvist et al., 2021).

However, traditional CBT-I is time-consuming and resource-intensive, and lack of qualified practitioners and high attrition rates may be barriers to its widespread implementation (Ellis et al., 2015). A more cost-effective way of providing treatment at scale is internet-based CBT-I, which is effective and more accessible (McLay et al., 2020). Given a choice, most adolescents chose internet-based CBT-I over face-to-face therapy indicating acceptability (de Bruin et al., 2015). However, engagement and adherence issues remain. For example, in Zetterqvist et al.’s (2021) internet-based CBT-I trial, course completion was low with participants attending an average of just over half the sessions. This is particularly concerning for an internet-based intervention aiming towards greater accessibility and reflects a trend in adolescent treatment in which drop-out and disengagement tend to be high (Cohen & Schleider, 2022). Thus, briefer treatment alternatives that do not necessitate repeat visits in order to benefit are needed (Schleider et al., 2020b).

Brief, single session interventions (SSIs) can be as effective as longer interventions (Schleider & Weisz, 2017). A SSI is intentionally designed as a stand-alone one-off intervention, without the need for a return visit to complete the course. SSIs are guided by a “disruptive innovation” model (Christensen et al., 2006) aiming to provide simpler, cost-effective, scalable interventions delivered through non-traditional means including digital technologies such as computers or smartphones (Schleider et al., 2020b). Results from a meta-analysis of 50 RCTs reported a significant beneficial effect of SSIs for youth mental health reflecting a “small-to-medium” overall effect (g = .32) that is comparable to traditional, multi-session, face-to-face treatment outcomes across disorders such as anxiety, conduct problems, depression, and general distress in multi-problem youths (Schleider & Weisz, 2017). Schleider et al.’s meta-analysis included mostly therapist-administered SSIs, and also 11 included studies tested self-administered SSIs (e.g., attention bias modification; “growth mindset” programs; self-affirmation interventions). Additionally, this meta-analysis included 13 treatment trials and 37 prevention trials. Self-administered digital SSIs are theoretically precise, ranging from 5-to-90-min, and designed to be completed in one sitting (Schleider et al., 2020b). A large recent trial indicated that they can reduce depression symptoms in adolescents at scale and in a pandemic context, including in those from under-served communities who do not tend to access traditional service provisions (Schleider et al., 2022). Thus, such digital SSIs may offer a novel, scalable, cost-effective approach to extending the provision of help for youth mental health, including for sleep problems—although the applicability of an SSI model to addressing sleep problems directly has not been formally assessed.

User experience (UX) plays a key role in ensuring that novel mental health interventions are optimized to support user engagement and completion (Lemon et al., 2020). Incorporating UX testing into the intervention development process can increase user satisfaction, improve treatment adherence, and facilitate greater empowerment of service users (Knowles et al., 2014). However, despite this, UX design has seldom been used in the process of designing new psychosocial interventions. This study thus adopts a more novel bottom-up approach to designing mental health interventions, focusing on UX design in the early stages of development and testing.

UX testing is based on principles of Design Thinking (DT) such as empathy towards the users’ needs, multidisciplinary ideation, and iterative development (Scholten & Granic, 2019). UX is also closely linked to UX Design theories and principles such as Nielsen’s Usability Heuristics (1994). These principles emphasise using language that is familiar to users, promoting user control and freedom, ensuring consistency and efficiency of use, emphasising recognition over recall, and creating aesthetic designs. UX principles are grounded in theories of cognitive psychology such as colour theory (Kimmons, 2020) and cognitive ergonomics (Turner, 2017). Recognition that UX may be influenced by factors beyond efficiency has also led to research into the emotional usability of interventions and emotional design (Kumar et al., 2019). The emotional design approach holds that designs that evoke positive emotional responses facilitate good user experience, which aligns with the process of user-centred design (Turner, 2017).

To address the need for accessible, scalable interventions for sleep problems in adolescents, a self-help online Sleep SSI has been developed by three paediatric psychology and sleep experts, ML, JH, and FO, drawing on evidence-based principles that have been tested extensively in evidence-based longer-term or briefer in-person interventions, such as Brief-Behavioral Treatment for Insomnia (BBTI; Gunn et al., 2019). The online SSIs developed and tested by Dr Jessica Schleider (e.g. growth mindset, behavioural activation SSIs) were used as a basis for developing the sleep SSI (Schleider et al., 2020a; Schleider et al., 2022; Schleider & Weisz, 2018). The Sleep SSI was developed using a user-centred design approach which involves the active participation of users in the development of the intervention (Zhang & Dong, 2009). Understanding user experiences of this intervention is thus essential to determine whether it meets the needs and preferences of adolescents experiencing sleep problems as a core part of its development. We herewith aimed to explore user experiences of a newly developed online Sleep SSI through retrospective think-aloud interviews to inform further intervention development.

## Methods

### Study design

We adopted a qualitative approach to gain an in-depth understanding of user experiences of the sleep SSI (Newton et al., 2021; Santesteban-Echarri et al., 2017). We used an exploratory cross-sectional qualitative design, including retrospective think-aloud interviewing techniques. Reflexive Thematic Analysis was used to analyse the data (Clarke & Braun, 2021). Ethical approval was obtained from the University of Bath Psychology Research Ethics Committee (Ref: UGM 22-027).

## Participants

Adolescents aged 16–19 years, living in the UK were eligible to participate, and they were recruited using a combination of purposive and convenience sampling methods (Clarke & Braun, 2021). Inclusion criteria included if they felt that they were experiencing sleep problems, could speak English at a conversational level, and confirmed that they were within the specified age range on the consent form (ages 16–19; see Procedure section for details on the recruitment process).

The final sample consisted of 11 participants, mean age 18.18 years (*SD* = .79, range 17–19). The participants were relatively diverse in identified ethnicity (54.5% Black/Black British, 36.4% Asian/Asian British, 9.1% White/White British) and gender (27.3% Male, 72.7% Female) (see Table 1). Pseudonyms have been used to ensure the confidentiality of the participants.

**Table 1.** Demographic characteristics of participants.

Participant	Age	Gender	Ethnic group
Zoe	19	Female	Asian/Asian British
Mel	18	Female	Black/Black British
Sam	18	Male	Black/Black British
Dan	18	Male	Black/Black British
Alina	18	Female	Black/Black British
Maya	17	Female	Asian/Asian British
Sara	17	Female	Asian/Asian British
Amy	19	Female	White/White British
Roy	18	Male	Black/Black British
Erin	19	Female	Black/Black British
Ida	19	Female	Asian/Asian British

## Materials

### Development of the intervention

To design this intervention, the collaborators drew on prior work by Orchard and colleagues (2020b) who developed a brief intervention for adolescents informed by CBT for Insomnia (e.g., de Bruin et al., 2018) as well as principles of Brief Behavioural Treatment for Insomnia (Kwon et al., 2021) and adolescent-specific interventions of the Transdiagnostic Intervention for Sleep and Circadian Dysfunction (Harvey et al., 2021). The treatment draws on components from these cognitive and behavioural interventions for insomnia, including sleep- and psycho-education, sleep hygiene, stimulus control (establishing regular schedules, associating bed with sleep, consolidating sleep to nighttime), and arousal reduction (e.g., wind down routines before bed to calm mind and body). Sleep restriction techniques are not included, due to the absence of direct clinical supervision to determine whether this is appropriate.

**Figure 1.**
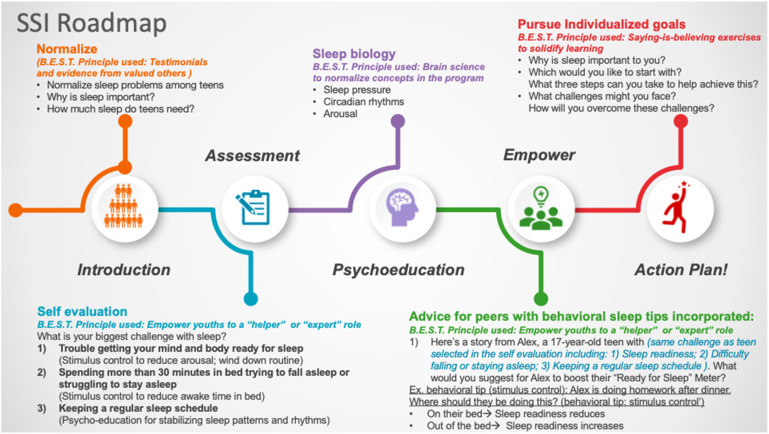
SSI roadmap.

To package this into a single session intervention, existing SSIs for youth mental health developed and trialled by Schleider et al. (2020a, 2020b) were used as a template and adolescents were involved in helping to present the intervention as accessible and interesting. This process included sharing preferences and perspectives for phrasing of the intervention, as well as colours and images that they found developmentally appropriate and engaging to be included in the intervention. Schleider et al. (2020a) suggested four primary elements based on qualities common to self-administered SSIs and brief interventions that have reduced psychopathology in youth to guide the development of SSIs for youth mental health known as “B.E.S.T.” elements. They stand for: B: Brain science to normalize concepts in the program; E: Empower youths to a “helper” or “expert” role; S: Saying-is-believing exercises to solidify learning; T: Testimonials and evidence from valued others. The current sleep SSI is based on the “B.E.S.T.” principles. This framework, drawing upon SSI principles and elements of social psychology, provides an example of a helpful guideline. Experts used the “B.E.S.T.” principles to co-create and refine the material, with design input from graduate students. Each component of the SSI has been informed by “B.E.S.T.” principles which are detailed in the SSI roadmap. Additionally, two advisory groups consisting of adolescents between ages 16–19 were recruited from local high schools and universities in the US and UK to give their input and feedback for intervention development purposes, laying the groundwork for a user-centred design. The steps involved in the Sleep SSI have been summarized in Figure 1. The prototype intervention within Qualtrics, which is the intended end user platform for administration, was used for this study.

### Semi-structured questions

Participants were asked some open-ended semi-structured questions on their overall experience of the intervention (see online supplementary materials S1. for topic guide). These questions were developed drawing on other studies that had used similar qualitative protocols to evaluate psychological interventions. We then used think-aloud techniques, which are useful in determining the acceptability and feasibility of online interventions (Davies et al., 2015; Yardley et al., 2015). Such interviews differ from cognitive interviews in that while cognitive interviews use targeted interview questions to evaluate participant comprehension during an activity, in think aloud interviews, participants are asked to freely talk out loud about their thoughts while completing a certain task (Davies, 2018). Certain pre-prepared prompts are also provided asking participants to verbalise their thoughts and feelings about the intervention (Tinner et al., 2020). There are two variations in think-aloud protocols; (1) concurrent approach in which participants are shown the materials for the first time during the interview and asked to think aloud as they look at them, and (2) retrospective approach, in which participants work through the materials independently and are then subsequently asked to recall their thoughts at a later time (Van Den Haak et al., 2003). While the concurrent think-aloud protocol provides insight into the participants’ thought processes in real-time, the process of thinking aloud may cause reactivity resulting in a poorer performance (Van Den Haak et al., 2003). On the other hand, the retrospective think-aloud protocol enables the participants to work through the intervention silently which more closely resembles regular working procedures (Van Den Haak et al., 2003). While such retrospection may lead to incomplete interpretation by the participants, forgetting and fabrication, and less reporting on the affective aspects of participants’ experiences, these shortcomings may be reduced by providing some kind of stimuli to participants to aid them in their retrospective verbalisations (Van Den Haak et al., 2003). Providing screen captures or video recordings to aid retrospection as has been done in this study may thus incorporate the benefits of concurrent methods into retrospective protocols (Van Den Haak et al., 2003). So that our participants experienced the sleep SSI as it is intended to be i.e. as self-help, we opted to use a retrospective think-aloud protocol, and asked the participants to go through the intervention prototype themselves, and then revisited it page by page by screen-sharing during the interviews to facilitate retrospective recall.

### Procedure

The study was advertised on the Department of Psychology Research Participation Scheme at the University of Bath and the MQ Participate website in June–July 2022. We received responses from 17 potential participants to the study advert, of which 11 met the inclusion criteria and attended the online interviews on Microsoft Teams. Informed consent was obtained from all participants. An online information sheet was sent prior to the meeting to provide participants with more details about the study before they consented to participate in the intervention and interviews. Participants were given an opportunity to ask questions at the start of the meeting and were invited to electronically sign a consent form on Qualtrics. Participants were then asked to complete the intervention independently in the absence of the interviewer, which took approximately 25 minutes (M = 23.78; SD = 9.07; Range = 8.03–38.08). Following completion, participants rejoined the Teams Meeting and online think-aloud interviews were conducted and audio-recorded using an external audio recorder. Interviews were approximately 35–50 minutes. Total duration of participation ranged from 60–75 minutes. Participants were awarded 2 credits through the Research Participation Scheme or offered £5 or an equivalent Amazon voucher for their participation.

### Data analysis

We undertook our analysis within the Big Q qualitative paradigm which emphasises collecting and analysing data in line with qualitative research values (Clarke & Braun, 2013). We adopted an ontological position of critical realism which postulates the existence of a true reality but holds that experiences of reality are inevitably mediated by language and culture (Maxwell, 2012). Accordingly, an epistemological framework of contextualism was assumed which recognises that human knowledge and experiences are socially and culturally situated (Guba & Lincoln, 1994). These positions enabled the analysis to reflect participants’ views and use them to inform intervention modifications, while also recognising the social situatedness of such views. Following recent criticisms of the data saturation model and its theoretical inconsistency with Reflexive Thematic Analysis (Braun & Clarke, 2021), Malterud et al. (2016) concept of ‘information power’ was used to estimate sample size. The concept of information power is based on the idea that the sample size should be based on the information the sample holds that is relevant to the study, and thus the more relevant information the sample holds, the lower the number of required participants. The information power of the sample and in turn the size of the sample would depend on the aims of the study, the sample specificity, the use of established theory, the quality of interview dialogue, and the data analysis strategy (Malterud et al., 2016). Considering the relatively narrow study aims; novice researcher; exploratory cross-case analysis; and available time and resources (Patton, 2002), a moderate sample size of 10–15 participants was proposed. However, keeping with the iterative nature of qualitative research (Sim et al., 2018), information power was continually assessed during data collection and a final sample of 11 participants (8 females, 3 males) was determined accordingly.

Interviews were audio-recorded and transcribed verbatim. Transcripts were anonymised at the point of transcription. Data was analysed using Reflexive Thematic Analysis (TA) (Braun & Clarke, 2006, 2021). A critical realist approach to TA was adopted taking a broad inductive orientation. However, the theoretical flexibility of TA enabled the analysis to be informed by interpretive accounts of the data and existing research literature (Clarke & Braun, 2021). The first author AM initially read and re-read the transcripts, coded these and developed and refined the themes. They were then shared and discussed with ML and VW, and then with the wider group during meetings, during which time the themes were refined. The analysis process has been summarized in Table 2.

**Table 2.** Process of reflexive thematic analysis.

Stage of thematic analysis	Description of stage	Output of stage
Familiarization with the Dataset	- Each transcript was read at least twice	Notes about initial insights and interesting aspects
Coding	- Sections of the transcripts that addressed the research question were assigned codes (primarily inductively) using the comments function on Microsoft Word	31 codes were finally generated from the data
	-Each transcript was coded twice (iterative)	
	- Several codes were modified and combined over the course of this stage	
Generating initial themes	-Codes were extracted into a table using the DocTools Word extension, and linked interview extracts were arranged according to their codes on Microsoft Excel	9 initial themes were generated from the data
	- Similar codes were combined to generate initial themes. Using mind-maps	
Developing and Reviewing themes	-Initial themes were merged and refined by reviewing them against the compiled data extracts	4 final themes were generated
	-Themes and subthemes were also reviewed against the entire dataset to ensure that they reflected the meanings conveyed by the participants	Thematic map was created
	-Central organising concept for each theme was identified	
Refining, Defining, and naming themes	- Themes and subthemes were refined, and the boundaries of the themes were identified	
	-Each theme was named, drawing on words used by the participants to make the theme names informative and memorable	
Writing the report	- Data extracts that best conveyed the essence of each theme were selected as illustrative quotes	

To ensure credibility and reflexivity, the researcher who led on the analysis (AM) kept a reflective diary, attended regular supervision sessions (with ML & VW), and adhered to the Standards for Reporting Qualitative Research Guidelines (O’Brien et al., 2014).

## Results

Participants were initially asked about their overall experience of the intervention using open-ended questions related to accessibility, usefulness, and likability (see online supplementary materials S1. for topic guide). All participants (*n* = 11) reported finding the Sleep SSI acceptable and likeable. Ten out of the eleven participants stated that they found the Sleep SSI useful. One participant indicated that she did not find it very useful as she was already aware of most of the information presented.

**Figure 2.**
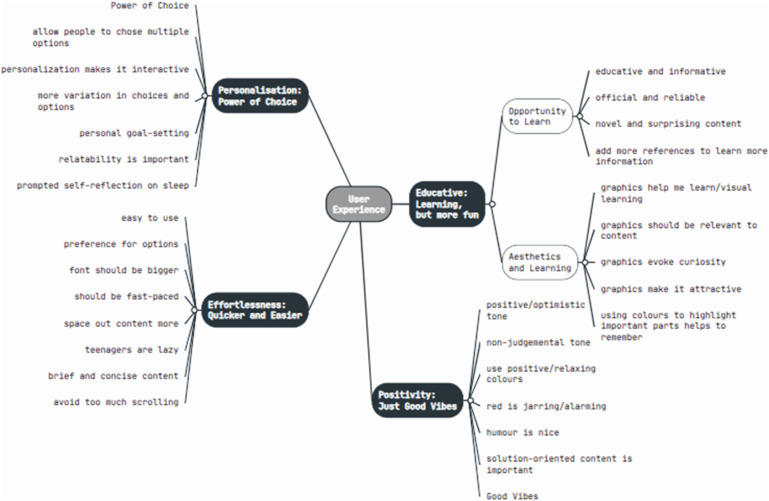
Final thematic map.

Four themes were generated from the analysis (see Figure 2 for thematic map), namely ‘Educative: Learning, but more fun’, ‘Effortless: Quicker and Easier’, ‘Personalization: Power of Choice’, and ‘Positivity: Just Good Vibes’. Each theme has been described below, with illustrative quotes.

### Theme 1 – Educative: Learning, but more fun

This theme encompasses the need to balance providing education and information and the importance of making the intervention appealing and fun. This theme has two subthemes – Opportunity to Learn, and Aesthetics and Learning.

#### Opportunity to learn

Participants found the intervention interesting and informative. They viewed the intervention as an opportunity to learn more about sleep and its impact on the mind and body, thus increasing their knowledge.

Cause I thought that not sleeping doesn’t affect anything, but now I’ve learned that if you don’t sleep, it affects your brain, how you think, how you do your work, how the body uses its energy (Dan, 18)

Participants also appreciated that the information provided was reliable and some suggested that more references could be added to enable them to learn more.

It’s referenced on the side corner of the page…Honestly, I found this such a nice detail that I would also have appreciated this on like the other slides as well. Just so that I can read up a bit more on all the information (Ida, 19)

#### Aesthetics and learning

Most participants appreciated the design of the intervention, and suggested that more relevant graphics, videos, and animations be added. Interestingly, these colours and animations not only made the intervention look more appealing, but also facilitated learning and helped make the educational and self-reflective aspects of the intervention more fun. However, participants indicated that it was important that the graphics be relevant and complement the content of the intervention.

I think having a video where you know I could just learn instead of having to read is just easier for me to absorb (Ida, 19)

The graphics here explaining these things. It’s attractive and it just helps explaining it to you. Like you’re learning but it’s more fun (Erin, 19)

Participants also indicated that highlighting important information in different colours was beneficial and suggested that more colours and visuals could be incorporated to help them learn better.

Another interesting thing could have been to have the whole page in a different colour. It would have maybe helped people remember all these points a bit better (Ida, 19)

### Theme 2 - Effortlessness: Quicker and easier

Both convenience and ease of use were important to participants undertaking the intervention. In particular, participants appreciated the brevity of content and stated that shorter sentences were easier to read and understand.

It was easy to do. I like the way it was brief and self-explanatory.…. Because we hate reading those long sentences (Mel, 18)

Participants preferred larger fonts, bigger pictures, clear instructions, and brief and concise information. Many participants also suggested that the graphics and font size be made bigger to make the process more effortless.

I think the font was quite small…having it being just more legible and easier to read would have eased the process and made it more mindless to do and just less effortful (Ida, 19)

Most participants also indicated a preference for selecting options rather than having to type into textboxes. Typing answers or even having to scroll down a lot was seen as tedious, and participants indicated that teenagers would prefer quicker and easier alternatives.

Options could have been given here to get people’s opinion. I think, a lot of young people wouldn’t just want to sit down and write things. Like options makes it more quicker and easier (Erin, 19)

With the scrolling, like I probably won’t read them all, having to scroll I would definitely ignore the last few…because I think teenagers are very lazy and we don’t really want to kind of scroll even if it is gonna be beneficial for us (Amy, 19)

This indicates that a more low-effort approach was desirable to adolescents, and many suggested that drop-down menus and options be provided alongside textboxes.

### Theme 3 – Personalization: Power of choice

The intervention offered participants information relevant to their own sleep problems based on the choices and options they selected. This ability to personalise the intervention was widely appreciated by participants and made the information more relatable and helpful in dealing with their specific sleep challenges. Furthermore, participants commented on how choices and options made the intervention more interactive and engaging.

It just gives you the ability to choose…The power of choice really comes into play here so it’s really perfect (Roy, 18)

I really like that it like allowed you to personalize what you wanted to learn about…like this was really, really helpful because it’s very relatable. (Zoe, 19)

This also helped the participants reflect on their own sleep and gave them more confidence in their ability to cope with sleep problems.

This kind of pushed me towards thinking of like small changes myself because it says that Riley felt overwhelmed…but then started with something very small…That gave me more hope about my sleep (Ida, 19)

Participants also suggested ways to make the intervention more personalized by providing greater variation in choices of sleep challenges, allowing them to pick multiple sleep problems, and providing options to learn more about the impact of poor sleep in chosen areas.

Maybe adding more problems that could be tackled to do with sleep. Because I feel like there are other problems people have….I think like personally, I struggle with getting consistent sleep like I wake up randomly, so maybe that (Sara, 17)

### Theme 4 – Positivity: Just good vibes

Positivity and a light-hearted optimistic tone were essential to making this intervention acceptable to adolescents. Participants indicated strong preferences for messages that were reassuring and less stressful.

I feel like a lot of people, especially teenagers, we don’t like things that stresses us out (Mel, 18)

In particular, participants appreciated that the content was encouraging and solution-oriented, as opposed to focusing on the negative consequences of poor sleep.

I like how it’s really positive in terms of actually convincing you like you can take control of your sleep…it’s not all-consumingly negative despite the negative impacts that sleep problems have (Zoe, 19)

This positive tone encouraged participants to re-evaluate their beliefs and perceptions of control regarding their own sleep.

Participants also commented favourably on the light-hearted humorous aspects of the intervention and found the pictures and animations uplifting and comforting. The theme of positivity thus also extended to the graphics and design of the intervention.

The graphic makes me like it more…I think the slide is just good vibes in general…and maybe use more positive colours….Like maybe green (Sara, 17)

I particularly liked the GIFs….a GIF came up on the next page correcting me and I thought that was quite funny because it softened the blow for getting something not perfectly correct (Ida, 19)

Both Sara and Ida highlight how graphics and colours play an integral role in conveying a positive optimistic tone and provide suggestions on how to improve this aspect of the intervention. Participants also found colours such as red alarming and preferred colours like green and blue which they found more positive and relaxing.

## Discussion

This is a qualitative exploration of the user experience of a newly developed self-help digital single session intervention (SSI) for adolescents who self-identify as struggling with sleep. Overall, the adolescents who took part in our think aloud interviews said they found the intervention helpful and useful. They particularly liked that it was educational but also fun, relatively low effort, and that there was the ability to choose what aspects of sleep to focus on. They also liked the positive focus of the intervention.

It is important to provide credible, reliable information in evidence-informed interventions such as the current SSI for sleep problems. Our participants appreciated the educational aspects of the intervention and perceived the content to be informative and reliable. Providing references to the source material aided their perception of credibility, and participants suggested that more references could be added so that they could learn more about the information provided on their own. This is supported by research suggesting that shaping knowledge around sleep by providing credible information may be an effective behaviour change technique (Arroyo & Zawadzki, 2022; Michie et al., 2015).

General principles about user design, and particularly the Aesthetic Usability principle, hold that visually appealing designs may boost usability and user engagement (Nielsen, 1994), i.e., “attractive things work better” (Norman, 2002 p. 36). Consistent with this, our participants preferred visual modes of learning and suggested that more graphics, animations, and colours be added to make the design more appealing. Research on other digital health interventions has also found that presenting interventions in an attractive and interactive way can improve their efficacy in adolescents (Hollis et al., 2017). The notion that graphics may improve learning aligns with the dual-coding theory which holds that pairing text with visual representations makes information easier to recall (Reed, 2012). Therefore, incorporating more references and visual elements may be avenues for further developing this intervention and other similar interventions.

Our participants favoured features that meant minimal effort was needed on their part, which highlights the important links between UX and cognitive ergonomics (i.e. the study of the cognitive processes involved in interactions between individuals and other systems such as technology, Zolotova & Giambattista, 2019). The capacity of the human working memory system is limited, and behaviour is often motivated by the urge to minimise effort (Zipf, 2016). These principles may underlie our participants’ preference for brief content, larger fonts, and audio-visual formats. As mobile devices have become more commonplace, it may be that small screens used to view online information also explain this preference. Our participants also preferred a list of pre-specified options to choose from rather than free textboxes, saying that they found these easier to use. Research on SSIs has reported high drop-out rates on pages where participants were asked to type out answers (Dobias et al., 2022). This mirrors another usability heuristic that emphasizes on recognition over recall (Nielsen, 1994). As giving options enabled participants to recognise rather than recall answers, they required less cognitive effort and may have thus facilitated better UX (Pereverseff & Bodner, 2020). Other research on digital mental health interventions has suggested that the high cognitive load demanded by them may be a barrier to their adoption by adolescents (Stiles-Shields et al., 2016). Thus, minimising the cognitive effort by, for example, providing pre-specified options as well as or instead of free textboxes may make this intervention more acceptable and engaging for adolescents. Part of the appeal of a single session intervention may be that it is briefer and easier to access compared to traditional, longer term interventions (Schleider et al., 2020b). Whilst completion rates for unguided digital mental health interventions are generally low as they require substantial mental and emotional effort during times of distress (Fleming et al., 2018), SSIs could be a potentially promising solution as they have been intentionally designed to lower time and effort demands, which may improve user engagement with interventions (Dobias et al., 2022).

Consistent with findings from several systematic reviews of digital mental health interventions (e.g. Borghouts et al., 2021), including in adolescents (e.g. Rasing et al., 2020), personalization features were liked by our participants. Even though the sleep SSI is self-directed, our participants appreciated being able to choose what they would like to learn more about and to have the subsequent intervention tailored accordingly. This aligns with research suggesting that adolescents value personal control when accessing mental health services (Plaistow et al., 2014). Personalization can also satisfy psychological needs such as autonomy in adolescents which may ensure future use of services (van der Velden et al., 2015). This also demonstrates a behavioural economics concept, the ‘IKEA effect’, which holds that offering users more choices for customisation provides greater opportunity for self-expression which can increase users’ retention of services (Ling et al., 2020). For adolescents who are essentially “digital natives”, ensuring that digital mental health interventions reflect the level of personalization and interactivity that they are accustomed to may thus be essential to making them more engaging and appealing (Knowles et al., 2014; Scholten & Granic, 2019).

It is also interesting that the positive tone, both in terms of the intervention content and its presentation visually, was important to participants. Adolescents appreciated the positive solution-oriented focus of the intervention and expressed dislike for content that was negative and stressful. As anxiety and stress may interfere with sleep (Alvaro et al., 2013), information focusing on the negative consequences of poor sleep can increase anxiety and worry about the consequences of not sleeping (Hiller et al., 2014). Thus, more encouraging content focusing on ways to improve sleep may be more helpful (Arroyo & Zawadzki, 2022). Participants also noted that this positivity helped them re-evaluate their perceived control over sleep, and this could be a candidate mechanism to explore in work in the future. Positivity and ‘good vibes’ were also expressed through the colours and graphics of the intervention, finding the graphics comforting and preferring positive colours such as green over colours like red that they perceived as alarming. Research on colour theory and positive aesthetics suggests that colours may impact attitudes and emotions (Kimmons, 2020). Cool colours such as blue or green typically evoke feelings of comfort (Kimmons, 2020), while colours like red are more arousing and often associated with danger (Elliot & Maier, 2007). There is a need for more in-depth exploration into the emotional usability of the sleep SSI and other similar interventions, focusing on participants’ feelings and emotions, to create a more positive, satisfying user experience (Marchitto & Canas, 2011).

## Strengths and limitations

Whereas digital mental health interventions are commonly tested on majority white populations (Valdez et al., 2012), this study included participants from diverse ethnic backgrounds, although the sample was predominantly female. This is important as ethnic minorities may be disproportionately affected by and less likely to seek help for sleep problems (Jackson et al., 2020), and SSIs have documented potential to reach these underserved populations (Schleider et al., 2020a). Another strength of this study lies in its user-centred design approach.

However, the sample size was small and recruited solely from the UK, and was not intended to be a representative sample. Participants were self-selecting and were also provided an information sheet about the study and then asked certain semi-structured questions prior to the think-aloud portion of the interviews, which could have influenced their experience of the intervention and their reporting of it. The use of retrospective think-aloud interviews may also have led to participants forgetting some of their initial thoughts about the intervention. Lastly, participants self-identified as experiencing sleep problems and no data was collected on the severity of such problems. Thus, it is not possible to determine how these factors may have shaped user experiences, and this remains an important area of future research. Furthermore, while this study found this sleep SSI to be acceptable to adolescents, further research is required to determine its effectiveness in addressing sleep problems in adolescents.

## Clinical implications

Promisingly, adolescents in our study found the newly developed sleep SSI acceptable, and it may hold promise as a means of providing a brief, scalable, accessible addition to traditional interventions. Building on this study, feedback will be incorporated and future work will aim to further develop, refine, and evaluate the effectiveness of the sleep SSI using rigorous research designs. There may also be the potential to use the sleep SSI as an adjunct to other clinical services addressing sleep problems or as a preventive measure for sleep or other mental health problems.

## Conclusion

Overall, our findings will inform our further developments of this Sleep SSI but are also applicable to other similar interventions for adolescents. This study also highlights the value of involving adolescents in the development of digital mental health interventions aimed at their cohort, and of using think-aloud interviews to explore their experience of user design. It is apparent that educational aspects, positivity, ease of use, and scope for personalization impacted user experience, and participants provided suggestions on how to improve these aspects. Next steps involve incorporating these suggestions into the intervention. Future research is also needed to determine how best to trial this intervention to determine feasibility and effectiveness among adolescents.

## Supplemental Material

Supplemental Material - How do adolescents experience a newly developed Online Single Session Sleep Intervention? A Think-Aloud Study Supplemental Material for How do adolescents experience a newly developed Online Single Session Sleep Intervention? A Think-Aloud Study by Ananya Maity, Angela W. Wang, Melissa J. Dreier, Vuokko Wallace, Faith Orchard, Jessica L. Schleider, Maria E. Loades, and Jessica L. Hamilton in Clinical Child Psychology and Psychiatry.
