# Supplementary material for: How do adolescents experience a newly developed Online Single Session Sleep Intervention? A Think-Aloud Study
Source: Clin Child Psychol Psychiatry. 2023 Nov 18;29(3):1137–58. doi: 10.1177/13591045231205475 (PMC11188559; doi:10.1177/13591045231205475)
Supplement: Supplemental Material - How do adolescents experience a newly developed Online Single Session Sleep Intervention? A Think-Aloud Study [file sj-pdf-1-ccp-10.1177_13591045231205475.pdf]

Online Supplementary Materials S1. Interview Schedule

Section 1: Overall Experience of the Intervention

1. How was your overall experience of using this intervention?

Prompts: What did you think about the content, design, and duration of this intervention? Which aspects of the intervention did you enjoy? Were there any things you would like to change?

2. How have your views on sleep changed following this intervention?

Prompt: How has this intervention affected your beliefs about your own sleeping habits?

3. How useful did you find this intervention?

Prompts: Which aspects of this intervention were most useful? Which areas needed improvement?

Section 2: Think-Aloud Interview

Main Prompt: As we go through the slides of the Online Single Session Sleep Intervention you just completed, try and think aloud on your thoughts, feelings, and experiences while completing these parts of the intervention?

Additional Prompts:

- What were your thoughts and feelings while completing this section?
- What did you think about the content on this page?
- What did you think about this question?
- What are your thoughts on the design of this section?
- What suggestions do you have to improve this section?
- How was your experience of using these free text boxes?

We are now nearing the end of this interview. Thank you for your participation. Is there anything else you'd like to share with me?
